# Lenvatinib for the Treatment of Radioiodine-Refractory Differentiated Thyroid Cancer: Treatment Optimization for Maximum Clinical Benefit

**DOI:** 10.1093/oncolo/oyac065

**Published:** 2022-04-28

**Authors:** Lori J Wirth, Cosimo Durante, Duncan J Topliss, Eric Winquist, Eyal Robenshtok, Hiroyuki Iwasaki, Markus Luster, Rossella Elisei, Sophie Leboulleux, Makoto Tahara

**Affiliations:** Harvard Medical School, Massachusetts General Hospital, Boston, MA, USA; Sapienza University of Rome, Rome, Italy; Alfred Health, and Monash University, Melbourne, Australia; University of Western Ontario, London, ON, Canada; Rabin Medical Center, Beilinson Hospital, Petach Tikva, Sackler Faculty of Medicine, Tel Aviv University, Israel; Kanagawa Cancer Center, Asahi-ku, Yokohama, Kanagawa, Japan; University Hospital Marburg, Marburg, Germany; University of Pisa, Lungarno Pacinotti, Pisa, Italy; Gustave-Roussy, Villejuif, France, and the University of Paris-Saclay, Gif-sur-Yvette, France; National Cancer Center Hospital East, Kashiwa, Japan

**Keywords:** differentiated thyroid cancer, lenvatinib, systemic therapy, toxicity, radioiodine refractory

## Abstract

**Background:**

Lenvatinib is a multitargeted tyrosine kinase inhibitor approved for treating patients with locally recurrent or metastatic progressive radioiodine-refractory differentiated thyroid cancer (RR-DTC). In this review, we discuss recent developments in the optimization of RR-DTC treatment with lenvatinib.

**Summary:**

Initiation of lenvatinib treatment before a worsening of Eastern Cooperative Oncology Group performance status and elevated neutrophil-to-lymphocyte ratio could benefit patients with progressive RR-DTC. The median duration of response with lenvatinib was inversely correlated with a smaller tumor burden, and prognosis was significantly worse in patients with a high tumor burden. An 18 mg/day starting dose of lenvatinib was not noninferior to 24 mg/day and had a comparable safety profile. Timely management of adverse events is crucial, as patients with shorter dose interruptions benefitted more from lenvatinib treatment. Caution should be exercised when initiating lenvatinib in patients who have tumor infiltration into the trachea or other organs, or certain histological subtypes of DTC, as these are risk factors for fistula formation or organ perforation. The **S**tudy of (**E**7080) **LE**nvatinib in Differentiated **C**ancer of the **T**hyroid (SELECT) eligibility criteria should be considered prior to initiating lenvatinib treatment.

**Conclusions:**

Current evidence indicates that patients benefit most from lenvatinib treatment that is initiated earlier in advanced disease when the disease burden is low. A starting dose of lenvatinib 24 mg/day, with dose modifications as required, yields better outcomes as compared to 18 mg/day. Appropriate supportive care, including timely identification of adverse events, is essential to manage toxicities associated with lenvatinib, avoid longer dose interruptions, and maximize efficacy.

## Teaser

Considering the difficulties associated with treating radioiodine-refractory differentiated thyroid cancer (RR-DTC) and the safety profile of tyrosine kinase inhibitors, a treatment plan needs to be carefully considered, to maximize efficacy while minimizing toxicity in patients with RR-DTC. This review highlights important developments of the past few years in the optimization of treatment of RR-DTC with lenvatinib.

**Implications for Practice**

Lenvatinib is approved for the treatment of patients with radioiodine-refractory differentiated thyroid cancer (RR-DTC), but as is true with other TKIs, it is associated with a variety of toxicities. To derive maximum clinical benefit from lenvatinib, clinicians must consider various factors such as timing of treatment initiation, optimal starting dose, risks associated with the treatment, and patient age. Therefore, an ongoing discussion regarding the optimization of lenvatinib treatment is essential to help clinicians make better decisions to improve the prognosis of patients with RR-DTC. In this review, we summarize the available literature regarding optimization of lenvatinib treatment in patients with RR-DTC.

## Introduction

Death due to thyroid cancer is relatively rare, with a mortality rate of approximately 0.5 deaths per 100 000 individuals.^1^ Differentiated thyroid cancer (DTC) is the most common type of thyroid cancer, and it accounts for approximately 95% of all thyroid cancer cases.^2^ DTC is usually asymptomatic and frequently is discovered incidentally.^3^ Distant metastases are present in <10% of patients with DTC, with about half of them detected at the initial diagnosis, and the remaining discovered during the follow-up period after initial treatment.^4^ Approximately 85% of patients are cured of DTC after treatment with surgery, radioiodine therapy, thyroid-stimulating hormone suppression, or a combination of these therapies.^5,6^ However, 5%-15% of patients with DTC have de novo resistance or become resistant to radioiodine, and they are categorized as having radioiodine-refractory DTC (RR-DTC).^5-8^ The 5-year survival rate for metastatic RR-DTC remains low at 10%, and treatment options are limited.^9^

Targeted tyrosine kinase inhibitors (TKIs)—including vascular endothelial growth factor receptor inhibitors—that lead to the inhibition of tumor cell growth pathways, have shown activity in the treatment of progressive RR-DTC.^2^ The National Comprehensive Cancer Network (NCCN) recommend lenvatinib or sorafenib (2 distinct TKIs) as systemic therapy for progressive and/or symptomatic RR-DTC, and RET inhibitors for patients with tumors harboring RET mutations.^10^ Sorafenib was approved for the treatment of RR-DTC based on results from the phase III DECISION trial, in which a 5-month improvement in progression-free survival (PFS) was observed.^11^

Lenvatinib is a multikinase inhibitor targeting vascular endothelial growth factor receptors 1-3, fibroblast growth factor receptors 1-4, platelet-derived growth factor receptor alpha, and RET and KIT proto-oncogenes.^12^ Lenvatinib was approved for the treatment of patients with locally recurrent or metastatic progressive RR-DTC based on results from the pivotal **S**tudy of (**E**7080) **LE**nvatinib in Differentiated **C**ancer of the **T**hyroid (SELECT).^12,13^ SELECT was a phase III, randomized, double-blind study that compared lenvatinib (*n* = 261) versus placebo (*n* = 131) in patients with RR-DTC.^13^ Lenvatinib was associated with significant improvement in PFS versus placebo (medians: 18.3 months [95% CI 15.1-not estimable] vs. 3.6 months [95% CI 2.2-3.7]; hazard ratio [HR], 0.21 [99% CI 0.14-0.31]; *P* < .001). The PFS benefit was maintained in patients with all reported histological subtypes of RR-DTC including papillary, poorly differentiated, follicular, and Hürthle cell; and in patients who had received 1 prior tyrosine kinase treatment.^13^ The response rate was also significantly improved with lenvatinib versus placebo (64.8% vs. 1.5%; odds ratio, 28.87 [95% CI 12.46-66.86]; *P < .*001).^13^ These findings were confirmed in an updated analysis of SELECT with a longer surveillance period: median PFS was longer in the lenvatinib group versus placebo (19.4 vs. 3.7 months; HR 0.24 [99% CI 0.17-0.35]; nominal *P < .*0001).^14^ Among patients treated with lenvatinib, median PFS in patients with complete or partial responses was 33.1 months [95% CI 27.8-44.6], whereas it was only 7.9 months [95% CI 5.8-10.7] in nonresponders.

Lenvatinib, like many TKIs, is associated with a variety of toxicities. In SELECT, the incidence of grade 3 or higher treatment-related adverse events (TRAEs) was 75.9% in the lenvatinib group and 9.9% in the placebo group.^13^ More patients in the lenvatinib group compared with the placebo group experienced treatment discontinuation due to adverse events (AEs) (14.2% vs. 2.3%), dose interruption (82.4% vs. 18.3%), or dose reduction (67.8% vs. 4.6%).^13^ The most common AEs that led to lenvatinib interruption or reduction were diarrhea (22.6%), hypertension (19.9%), proteinuria (18.8%), and decreased appetite (18.0%).

Treatment options are limited for patients with progressive RR-DTC. However, given the toxicity profile of TKIs,^11,13,15^ clinicians need to give special consideration to the timing of systemic treatment initiation. Although overtreatment is a possibility, undertreatment may lead to significant symptoms and shortening of life. To balance over/under-treatment of DTC,^15^ it is important to identify predictive and prognostic biomarkers for advanced DTC.^16^ Moreover, in patients with slowly progressive disease, the conservative approach of active surveillance may be used to avoid overtreatment; however, identifying patients suitable for active surveillance remains a challenge for clinicians.^17^

Given the difficulties associated with treating RR-DTC and the safety profile of TKIs, including lenvatinib, it is critical for clinicians to devise a treatment plan to maximize efficacy while minimizing toxicity in patients with RR-DTC. This balance can be achieved by ensuring that lenvatinib is initiated at a suitable starting dose at the right time during disease progression, and that treatment is maintained with adequate and rapid management of any toxicities. Herein, we review important developments from the past few years in the optimization of treatment of RR-DTC with lenvatinib.

## Materials and Methods

The PUBMED database was searched using the following search terms: VEGF inhibitors, lenvatinib, RR-DTC, thyroid cancer, starting dose, dose interruptions, tumor burden, and fistulas, in the English language. The reference lists of selected articles were screened for additional relevant studies. Data from eligible studies were extracted and reviewed by the authors.

## Review

### Earlier Initiation of Lenvatinib May Lead to Better Clinical Outcomes

The timing of initiation of systemic therapy is one of the biggest challenges clinicians face when treating patients with RR-DTC. For patients with RR-DTC who are asymptomatic and have pulmonary nodules that are small (< 1 cm) and unchanging or slowly progressive (doubling every 5 years), a “watch and wait” approach can be used as in the short-term they tend to have a good quality of life.^18,19^ However, it is crucial to monitor the disease carefully as progression may occur before patients become symptomatic.^19^ Based on recommendations from individual guidelines, there is no strong consensus about the timing for initiation of systemic therapy in patients with RR-DTC.^4,10,20-22^ In general, for patients who are symptomatic, have lesions > 1 cm in size, or are progressing rapidly (doubling in 2-3 years), initiation of systemic therapy should be considered. The decision to initiate systemic therapy should be made in the context of a multidisciplinary team including endocrinologists and oncologists, and should take into account tumor parameters and clinicopathological characteristics of the patient.^18,19^

The Eastern Cooperative Oncology Group performance status (ECOG PS; a measure of patient’s level of functioning) and elevated neutrophil-to-lymphocyte ratio (NLR) are prognostic factors associated with survival and response to therapy in several cancer types.^23,24^ Similarly, tumor burden has also been studied as a prognostic indicator in patients with RR-DTC.^25^ Key findings from several studies discussed herein are presented in Table 1.^14,25-29^

**Table 1.** Optimal lenvatinib treatment for patients with radioiodine-refractory differentiated thyroid cancer.

	ECOG PS and NLR	Lower tumor burden	Lung metastases ≥ 1 cm	Appropriate starting dose	Dose interruptions
**Studies**	Taylor et al^26^	Gianoukakis et al^14^ Suzuki et al^25^	Tahara et al^27^	Brose et al ^28^	Tahara et al^29^
**Key findings**	Patients with lower ECOG PS and lower NLR values at baseline had improved outcomes with lenvatinib treatment	The median duration of response with lenvatinib treatment was inversely correlated with a smaller tumor burden. Among patients with RR-DTC treated with lenvatinib, prognosis was significantly worse in patients with a high tumor burden	OS and PFS were significantly prolonged with lenvatinib versus placebo in patients who had baseline lung metastases of ≥1 cm, even though 89% of patients in the placebo arm crossed over to the lenvatinib arm later in the study	A lower starting dose of lenvatinib (18 mg/day) was not noninferior to the approved starting dose (24 mg/day), and safety profiles were comparable for the 2 doses	ORR was higher and a greater PFS benefit was obtained with lenvatinib treatment versus placebo in the group of patients with shorter dose interruptions compared with the group with longer dose interruptions
**Take-home message**	Initiation of lenvatinib treatment before a worsening in ECOG PS and NLR could be beneficial for patients with progressive RR-DTC	In patients with RR-DTC, early initiation of lenvatinib treatment when the tumor burden is lower may have maximum clinical benefit	Delaying initiation of lenvatinib treatment may negatively impact prognosis in patients with lung metastases ≥ 1 cm	The approved 24-mg starting dose of lenvatinib, with dose modifications as required, is the best treatment strategy for maximum clinical benefit in RR-DTC	Timely and proactive management of toxicities is essential to avoid longer dose interruptions when treating patients with RR-DTC with lenvatinib

Abbreviations: ECOG PS, Eastern Cooperative Oncology Group performance status; NLR, neutrophil-to-lymphocyte ratio; ORR, objective response rate; OS, overall survival; PFS, progression-free survival; RR-DTC, radioiodine-refractory differentiated thyroid cancer.

An exploratory post hoc analysis of SELECT assessed baseline ECOG PS and NLR as prognostic markers in patients with RR-DTC treated with lenvatinib.^26^ It was observed that patients treated with lenvatinib with a baseline ECOG PS of 0 had improved PFS (HR 0.52 [95% CI 0.35-0.77]; nominal *P = .*001) and overall survival (OS) (HR 0.42 [95% CI 0.26-0.69]; nominal *P = .*0004) compared with patients with a baseline ECOG PS of 1. Moreover, objective response rate (ORR) was also improved in patients with ECOG PS 0 at baseline (78.5% [95% CI 71.8-85.2]) versus patients with ECOG PS 1 (51.0% [95% CI 41.4-60.6]). Similarly, patients with an NLR ≤ 3 had improved PFS (HR 0.43 [95% CI 0.29-0.65]; nominal *P < .*0001) and OS (HR 0.48 [95% CI 0.29-0.78]; nominal *P = .*0029) compared with patients with an NLR > 3. The results from this study indicate that initiation of lenvatinib treatment before a worsening in ECOG PS and NLR might maximize treatment efficacy for patients with progressive RR-DTC. Although it may be argued that this analysis was fraught with lead-time bias (ie, patients with lower ECOG PS and NLR values had an earlier diagnosis of their disease, and therefore their OS was improved), it is important to note that efficacy measures unaffected by lead-time bias such as ORR were also improved in patients with lower ECOG PS.

Several additional analyses (Table 1) have further demonstrated the importance of early initiation of treatment with lenvatinib. In an updated analysis of SELECT (with a later data cutoff date), median duration of response (DOR) with lenvatinib was inversely correlated with a smaller tumor burden.^14^ Specifically, median DORs were 44.3, 27.5, 18.0, and 15.7 months for patients with tumor sizes of ≤ 35 mm, 35-60 mm, 60-92 mm and > 92 mm, respectively. In addition, a retrospective review of clinical records from a small population of patients with RR-DTC treated with lenvatinib found that prognosis was significantly worse in patients with a high tumor burden.^25^ In a post hoc analysis of patients with lung metastases from SELECT^27^ (Table 1), OS and PFS were significantly prolonged with lenvatinib treatment versus placebo in patients who had baseline lung metastases as small as 1.0 cm, despite the fact that 89% of patients with lung metastases ≥ 1.0 cm from the placebo arm crossed over to the lenvatinib treatment arm. As such, the survival benefit with lenvatinib treatment, despite the high crossover rate, suggests that delaying lenvatinib initiation may worsen the prognosis of patients with progressive RR-DTC and lung metastases ≥ 1.0 cm.

Caution should be used as several of the analyses described were conducted post hoc. However, taken together, earlier initiation of lenvatinib treatment in patients with RR-DTC (ie, among patients with a lower disease burden) appears to lead to more favorable long-term outcomes.

### Optimal Lenvatinib Starting Dose for Patients with RR-DTC

Lenvatinib is approved for the treatment of RR-DTC at a starting dose of 24 mg/day.^12^ In other indications, such as unresectable hepatocellular carcinoma, lenvatinib monotherapy is approved at a lower starting dose of 8 or 12 mg/day, based on body weight (< 60 kg or ≥ 60 kg, respectively).^12^ Given the toxicities associated with lenvatinib and the effectiveness of lenvatinib at lower starting doses for other indications, the authors have noted that some clinicians prefer to begin treatment of RR-DTC at a lower dose. A population pharmacokinetics/pharmacodynamics modeling analysis simulated the testing of 7 lenvatinib dosing regimens in patients with RR-DTC.^30^ The results supported the decision of clinicians to start lenvatinib at a lower dose, as lenvatinib 18 mg/day without up-titration was potentially found to provide comparable efficacy with a more favorable safety profile compared with a 24 mg/day starting dose.^30^

As such, a multicenter, randomized trial was performed to determine if a lower starting dose of lenvatinib (18 mg/day) could provide noninferior efficacy to the approved 24 mg/day starting dose while having an overall improved safety profile^28^ (Table 1). The ORR at week 24 was 57.3% (95% CI 46.1-68.5) in the 24-mg arm versus 40.3% (95% CI 29.3-51.2) in the 18-mg arm (odds ratio 0.50 [95% CI 0.26-0.96]). Moreover, as of week 24, incidences of grade ≥ 3 treatment-emergent adverse events (TEAEs) were similar between the lenvatinib 18-mg arm (57.1%) compared with patients in the lenvatinib 24-mg arm (61.3%). Taken together, the results of this randomized study indicate that the approved lenvatinib 24 mg starting dose, with dose modifications as required, is the preferred treatment strategy for maximum clinical benefit in RR-DTC.

### Importance of Prompt and Proactive Supportive Care After Lenvatinib Treatment Initiation

In SELECT, 82.4% of patients in the lenvatinib treatment group had a dose interruption to manage toxicity; the mean lenvatinib dose was 17.2 mg/day, even though the planned starting dose was 24 mg/day.^13^ Although dose interruptions are a common method to alleviate AEs, there is concern that longer dose interruptions could potentially correlate with disease progression, as there is a possibility of tumor regrowth during the periods of dose interruption.^29^ The impact of prolonged dose interruptions on the efficacy of lenvatinib is not clear. To investigate the potential impact of dose interruptions, a post hoc analysis of SELECT was conducted in patients who received lenvatinib. Patients were divided into 2 groups based on their dose interruption: a shorter dose interruption group in which lenvatinib was interrupted for <10% of total treatment duration; and a longer dose interruption group in which lenvatinib was interrupted for ≥10% of the total treatment duration.^29^ The ORR was higher in patients in the shorter dose interruption group (76.1%) compared with patients in the longer dose interruption group (52.8%). Moreover, in a multivariate analysis of these data, a shorter dose interruption was associated with a longer PFS (HR 0.467 [95% CI 0.307-0.712]; nominal *P < .*0004; Table 1). Notably, the overall median dose intensity was higher in the shorter dose interruption group compared to the longer dose interruption group (20.1 vs. 14.6 mg/day/patient), which could have led to better outcomes in this group.

The study results of Tahara et al indicate that it is crucial for clinicians to initiate timely, appropriate, and proactive management of toxicities associated with lenvatinib to avoid longer dose interruptions.^29^ In patients with RR-DTC, the toxicity profile associated with lenvatinib is highly predictable, and several studies provide practical recommendations for managing the most common AEs observed, including, but not limited to, hypertension, diarrhea, fatigue/asthenia, decreased appetite, and decreased weight.^31,32^ Prophylaxis, regular monitoring, and management of symptoms are key to ensure that patients remain on the optimal dose of lenvatinib.^32^ In essence, any AE should be identified, graded, and managed through judicious dose interruptions and reductions, with concomitant care as necessary [Tables 2 and 3]. It is also important to educate clinicians and patients on recognizing lenvatinib-associated toxicities so that they can be addressed as soon as they appear.^33^

**Table 2.** Recommended management of selected treatment-related adverse events with lenvatinib treatment.

Treatment-related adverse event	Incidence in SELECT(%)^13^	Recommended management strategies^12,13,31,32^
Hypertension	67.8	• Grades 1-2: treat with antihypertensive agents without discontinuing lenvatinib. Dose reduction not necessary unless antihypertensive treatments do not control blood pressure • Grade 3: treat with antihypertensive agents and lenvatinib dose interruption. Lenvatinib can be resumed when hypertension is at grade ≤ 2 • Grade 4: discontinue lenvatinib treatment
Diarrhea	59.4	• Promptly manage with antidiarrheals and maintain patient hydration • Grade 3: lenvatinib therapy can be interrupted then resumed at lower doses upon management of diarrhea • Grade 4: discontinue lenvatinib
Fatigue or asthenia	59.0	• Recommend healthy and active lifestyle including aerobic and non-aerobic exercise • Monitor thyroid-stimulating hormone and hemoglobin levels • If fatigue becomes disabling, discontinue lenvatinib
Decreased appetite	50.2	• Refer patient to a dietitian or specialist nurse • Recommend high-calorie foods
Decreased weight	46.4	• If patient loses 10% of their baseline weight, interrupt treatment for 1 week, then resume at same dose • If weight loss reoccurs, interrupt treatment again
Proteinuria	31.0	• Grades 2–3: (1 to >3.5 g/24 h): consider dose interruption and refer to a nephrologist • Grade 4: discontinue lenvatinib
Gastrointestinal fistula^a^	1.5	• Monitor signs such as abdominal pain • Grades 3-4 fistula (or any grade gastrointestinal perforation): Discontinue lenvatinib treatment

Gastrointestinal fistula was an adverse event of special interest in SELECT.

**Table 3.** Dose modifications of lenvatinib recommended to mitigate adverse events, as recommended by the lenvatinib prescribing information.^12^

Dose interruption	Upon incidence of intolerable grade 2 or 3 AE, withhold lenvatinib until AE improves to grade ≤1, then resume at lower dose
Dose reduction	First dose reduction to 20 mg/day Second dose reduction to 14 mg/day Third dose reduction to 10 mg/day
Dose discontinuation	Discontinue lenvatinib upon incidence of grade 4 AE

Abbreviation: AE, adverse event.

### Risk of Developing Fistulas or Organ Perforation During Lenvatinib Treatment

Fistula formation and organ perforation are rare but life-threatening side effects associated with TKI therapy, including treatment with lenvatinib.^34,35^ In SELECT, gastrointestinal fistula formation occurred in 1.5% of patients treated with lenvatinib.^13^ Radiation therapy, prior surgery, and large thoracic tumor burden are risk factors for fistula formation associated with lenvatinib and other TKIs.^12,31,34,36^ In a recently published study, an analysis was performed to evaluate the prevalence of fistula and/or organ perforations in 95 patients with RR-DTC treated with lenvatinib at a single center.^35^ Potential risk factors for these severe AEs were also assessed. In this study, during treatment with lenvatinib, 14 patients (14.7%) developed a fistula or organ perforation, and more than half of these patients had infiltration of the trachea, bronchus, esophagus, pleura, or bladder at the time of starting treatment (or 6 months after starting treatment in the case of bladder infiltration). Based on a risk-factor analysis performed between the patients who did or did not develop these severe AEs, the researchers concluded that the presence of tumor infiltration and tumor histology (papillary and poorly differentiated) were significantly correlated with fistulas or organ perforation, while external beam radiation therapy (indication and total dose), lenvatinib starting dose, and duration of treatment were not significantly correlated. Therefore, clinicians should use their discretion and be vigilant for symptoms when initiating lenvatinib in patients who have tumor infiltration or certain histological subtypes of DTC.

It is important to note that there are case reports of the successful use of lenvatinib in a neo-adjuvant setting for the treatment of advanced unresected DTC with invasion of surrounding organs and no prior radioiodine therapy.^37,38^ As VEGFR TKIs are also associated with an increased bleeding risk,^39^ patients at risk for these complications need to be treated with caution or may need to explore nonantiangiogenic targeted therapies.

### Effect of Patient Age on Lenvatinib Treatment Benefit

A prespecified subanalysis of SELECT suggested that OS was significantly improved in older patients (aged > 65 years) treated with lenvatinib versus placebo (HR, 0.53 [95% CI 0.31-0.91]; *P = .*02), however, it is important to note that in this analysis, there were fewer OS events in younger patients (aged ≤ 65 years) and that survival data were not mature for these patients.^40^ Further, among placebo-treated patients, OS was significantly longer in patients aged ≤ 65 years compared with patients aged > 65 years (HR, 0.48 [95% CI 0.27-0.85]; *P = .*01), suggesting that delaying treatment may worsen the prognosis of older patients.^40^ A separate multivariate analysis showed that among patients from SELECT who had baseline lung metastases of ≥1.0 cm and were treated with lenvatinib, younger patients (aged ≤ 65 years) had a greater OS benefit (nominal *P = .*0243) compared with older patients (aged > 65 years).^27^ These results suggest that lenvatinib may yield a greater treatment benefit in younger patients.

## Discussion

The introduction of lenvatinib and sorafenib, oral multitargeted TKI systemic therapies, has dramatically altered the therapeutic landscape for patients with RR-DTC.^15^ Although there have been no trials conducted that directly compare lenvatinib to sorafenib in patients with RR-DTC,^15^ the NCCN guidelines have designated lenvatinib as the preferred systemic treatment for progressive and/or symptomatic RR-DTC.^10^ In SELECT, lenvatinib improved outcomes in RR-DTC versus placebo.^13^ Moreover, the efficacy of lenvatinib was maintained across various categories of radioiodine refractoriness that included no radioiodine uptake, disease progression within 12 months of radioiodine therapy, and extensive cumulative radioiodine exposure.^41^

Despite the efficacy of lenvatinib in SELECT, its toxic effects were considerable.^13^ While toxicity was generally manageable with standard clinical interventions or dose modifications, 14.2% of patients in the lenvatinib group discontinued treatment. Given the significant toxicity associated with lenvatinib, clinicians may hesitate to initiate treatment early in the disease, or they may start patients on a lower dose. Considering these findings, ongoing discussion regarding the optimization of lenvatinib treatment, especially in terms of starting dose and timing of initiation, are essential for improved patient prognosis.

In this review article, we highlight the results of several post hoc analyses of SELECT, as well as several real-world-data studies. Although the post hoc nature of many of these analyses is an inherent limitation when interpreting the results, these studies provide insights into strategies for optimizing lenvatinib treatment in patients with RR-DTC. In general, the post hoc analyses of ECOG PS, NLR, and lung metastases suggest that earlier intervention improves treatment outcomes of DTC.^26,27^

A starting dose of lenvatinib 18 mg/day was not noninferior to lenvatinib 24 mg/day, and the safety profile of both starting doses was comparable in highly selected patients within a randomized trial.^28^ As such, it is suggested that lenvatinib should be given at the approved starting dose and managed appropriately to avoid prolonged dose interruptions.^29^ However, in real-world experience with patients who are not suitable for a trial, have lower body weight, or are elderly, the 24 mg/day starting dose of lenvatinib could lead to toxicity and refusal by patients to resume treatment despite dose modifications. Therefore, clinicians must use their judgement in selecting a starting dose, but their decisions should be informed by the evidence that a 24 mg/day starting dose led to better outcomes in a clinical trial setting.^28^ Active monitoring and management of adverse events instituted at drug initiation, and an immediate response by the managing clinician to an adverse event report, may aid in improving patient compliance with treatment. An interesting topic that needs further research is the question of decrease of dosage versus short-term dose interruptions, and which of these strategies could be more beneficial for long-term patient outcomes. Considering the interpatient variability of exposure with lenvatinib, dose individualization of lenvatinib through therapeutic drug monitoring is also a promising area of research that could help minimize unacceptable AEs and provide maximum benefit to patients.^42,43^

The findings presented here may be valuable to clinicians when weighing the choice of initiating lenvatinib treatment; however, the impact of factors such as sex, body mass index, and glomerular filtration should also be considered. Other factors that may influence the decision to initiate lenvatinib treatment include tumor growth rate, tumor-related symptoms, and comorbidities. Caution should be exercised when initiating lenvatinib in patients with tumor infiltration of vital organs, as they may be at a higher risk for formation of fistulas and organ perforations.^35^ Disease sites are also an important consideration: for example, brain metastases, pleural effusion, and bone metastases bode a worse prognosis^44-46^ and may warrant rapid initiation of lenvatinib or initiation of local treatments for these metastases. Lastly, when initiating lenvatinib treatment, clinicians should consider the eligibility criteria for SELECT, which included measurable disease with progression according to Response Evaluation Criteria In Solid Tumors version 1.1 within 12 months after radioactive iodine therapy (despite radioactive iodine avidity at the time of treatment).^13^

## Conclusion

For patients with progressive RR-DTC, systemic therapy including lenvatinib is an essential therapeutic tool. It is crucial that lenvatinib treatment is optimized to gain maximum clinical benefit for patients. Data from several studies indicate that patients derive the most benefit from lenvatinib treatment that is initiated earlier in advanced disease, when the tumor burden is low. The optimal starting dose of lenvatinib for treatment of RR-DTC is 24 mg/day, with dose modifications as required. Prompt and appropriate supportive care is essential to manage toxicities associated with lenvatinib to minimize dose interruptions and maximize efficacy. Future directions for lenvatinib in the treatment of patients with RR-DTC include the ongoing phase II study of lenvatinib plus the immune checkpoint inhibitor pembrolizumab (NCT02973997). This trial aims to assess efficacy and safety of lenvatinib plus pembrolizumab in patients who had not received prior treatment with a VEGFR active multikinase inhibitor compared with patients who had pembrolizumab added to their treatment after they experienced progressive disease on lenvatinib alone. The combination of lenvatinib plus pembrolizumab has shown efficacy in other indications, specifically endometrial carcinoma and renal cell carcinoma,^47,48^ and represents an intriguing potential treatment for patients with RR-DTC.

## Data Availability

The data underlying this article will be shared on reasonable request to the corresponding author.
